# Conceptualising natural and quasi experiments in public health

**DOI:** 10.1186/s12874-021-01224-x

**Published:** 2021-02-11

**Authors:** Frank de Vocht, Srinivasa Vittal Katikireddi, Cheryl McQuire, Kate Tilling, Matthew Hickman, Peter Craig

**Affiliations:** 1 Population Health Sciences, Bristol Medical School, University of Bristol, Canynge Hall, 39 Whatley Road, Bristol, BS8 2PS UK; 2 NIHR School for Public Health Research, Newcastle, UK; 3 NIHR Applied Research Collaboration West, Bristol, UK; 4 MRC/CSO Social and Public Health Sciences Unit, University of Glasgow, Bristol, UK; 5 MRC IEU, University of Bristol, Bristol, UK

**Keywords:** Public health, Public health policy, Natural experiments, Quasi experiments, Evaluations

## Abstract

**Background:**

Natural or quasi experiments are appealing for public health research because they enable the evaluation of events or interventions that are difficult or impossible to manipulate experimentally, such as many policy and health system reforms. However, there remains ambiguity in the literature about their definition and how they differ from randomized controlled experiments and from other observational designs. We conceptualise natural experiments in the context of public health evaluations and align the study design to the Target Trial Framework.

**Methods:**

A literature search was conducted, and key methodological papers were used to develop this work. Peer-reviewed papers were supplemented by grey literature.

**Results:**

Natural experiment studies (NES) combine features of experiments and non-experiments. They differ from planned experiments, such as randomized controlled trials, in that exposure allocation is not controlled by researchers. They differ from other observational designs in that they evaluate the impact of events or process that leads to differences in exposure. As a result they are, in theory, less susceptible to bias than other observational study designs. Importantly, causal inference relies heavily on the assumption that exposure allocation can be considered ‘as-if randomized’. The target trial framework provides a systematic basis for evaluating this assumption and the other design elements that underpin the causal claims that can be made from NES.

**Conclusions:**

NES should be considered a type of study design rather than a set of tools for analyses of non-randomized interventions. Alignment of NES to the Target Trial framework will clarify the strength of evidence underpinning claims about the effectiveness of public health interventions.

**Supplementary Information:**

The online version contains supplementary material available at 10.1186/s12874-021-01224-x.

## Background

When designing a study to estimate the causal effect of an intervention, the experiment (particularly the randomised controlled trial (RCT) is generally considered to be the least susceptible to bias. A defining feature of the experiment is that the researcher controls the assignment of the treatment or exposure. If properly conducted, random assignment balances unmeasured confounders in expectation between the intervention and control groups*.* In many evaluations of public health interventions, however, it is not possible to conduct randomised experiments. Instead, standard observational epidemiological study designs have traditionally been used. These are known to be susceptible to unmeasured confounding.

Natural experimental studies (NES) have become popular as an alternative evaluation design in public health research, as they have distinct benefits over traditional designs [1]. In NES, although the allocation and dosage of treatment or exposure are not under the control of the researcher, they are expected to be unrelated to other factors that cause the outcome of interest [2–5]. Such studies can provide strong causal information in complex real-world situations, and can generate effect sizes close to the causal estimates from RCTs [6–8]. The term natural experiment study is sometimes used synonymously with quasi-experiment; a much broader term that can also refer to researcher-led but non-randomised experiments. In this paper we argue for a clearer conceptualisation of natural experiment studies in public health research, and present a framework to improve their design and reporting and facilitate assessment of causal claims.

Natural and quasi-experiments have a long history of use for evaluations of public health interventions. One of the earliest and best-known examples is the case of ‘Dr John Snow and the Broad Street pump’ [9]. In this study, cholera deaths were significantly lower among residents served by the Lambeth water company, which had moved its intake pipe to an upstream location of the Thames following an earlier outbreak, compared to those served by the Southwark and Vauxhall water company, who did not move their intake pipe. Since houses in the study area were serviced by either company in an essentially random manner, this natural experiment provided strong evidence that cholera was transmitted through water [10].

### Natural and quasi experiments

Natural and quasi experiments are appealing because they enable the evaluation of changes to a system that are difficult or impossible to manipulate experimentally. These include, for example, large events, pandemics and policy changes [7, 11]. They also allow for retrospective evaluation when the opportunity for a trial has passed [12]. They offer benefits over standard observational studies because they exploit variation in exposure that arises from an exogenous (*i.e.* not caused by other factors in the analytic model [1]) event or intervention. This aligns them to the ‘*do*-operator’ in the work of Pearl [13]. Quasi experiments (QES) and NES thus combine features of experiments (exogenous exposure) and non-experiments (observations without a researcher-controlled intervention). As a result, they are generally less susceptible to confounding than many other observational study designs [14]. However, a common critique of QES and NES is that because the processes producing variation in exposure are outside the control of the research team, there is uncertainty as to whether confounding has been sufficiently minimized or avoided [7]. For example, a QES of the impact of a voluntary change by a fast food chain to label its menus with information on calories on subsequent purchasing of calories [15]. Unmeasured differences in the populations that visit that particular chain compared to other fast-food choices could lead to residual confounding.

**Fig. 1:**
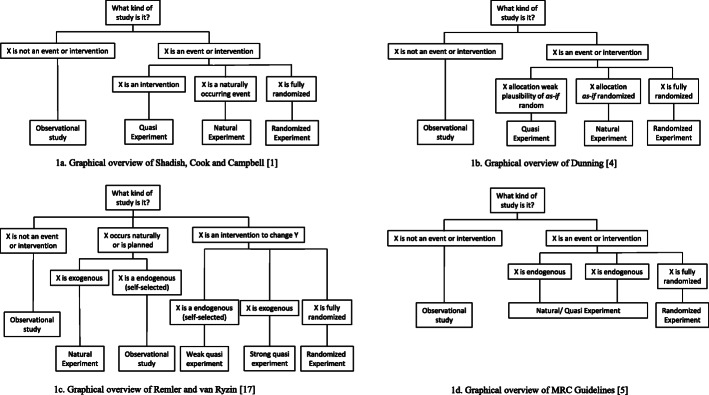
Different conceptualisations of natural and quasi experiments within wider evaluation frameworks

A distinction is sometimes made between QES and NES. The term ‘natural experiment’ has traditionally referred to the occurrence of an event with a natural cause; a ‘force of nature‘(Fig. 1a) [1]. These make for some of the most compelling studies of causation from non-randomised experiments. For example, the Canterbury earthquakes in 2010–2011 have been used to study the causal impact of such disasters because about half of an established birth cohort lived in the affected area with the remainder of the cohort living elsewhere [16]. More recently, the use of the term ‘natural’ has been understood more broadly as an event which did not involve the deliberate manipulation of exposure for research purposes (for example a policy change), even if human agency was involved [17]. Compared to natural experiments in QES the research team may be able to influence exposure allocation, even if the event or exposure itself is not under their full control; for example in a phased roll out of a policy [18]. A well-known example of a natural experiment is the “Dutch Hunger Winter” summarised by Lumey et al. [19]. During this period in the Second World War the German authorities blocked all food supplies to the occupied West of the Netherlands, which resulted in widespread starvation. Food supplies were restored immediately after the country was liberated, so the exposure was sharply defined by time as well as place. Because there was sufficient food in the occupied and liberated areas of the Netherlands before and after the Hunger Winter, exposure to famine occurred based on an individual’s time and place (of birth) only. Similar examples of such ‘political’ natural experiment studies are the study of the impact of China’s Great Famine [20] and the ‘special period’ in Cuba’s history following the collapse of the Soviet Union and the imposition of a US blockade [21]. NES that describe the evaluation of an event which did not involve the deliberate manipulation of an exposure but involved human agency, such as the impact of a new policy, are the mainstay of ‘natural experimental research’ in public health, and the term NES has become increasingly popular to indicate any quasi-experimental design (although it has not completely replaced it).

Dunning takes the distinction of a NES further. He defines a NES as a QES where knowledge about the exposure allocation process provides a strong argument that allocation, although not deliberately manipulated by the researcher, is essentially random. This concept is referred to as ‘as-if randomization’ (Fig. 1b) [4, 8, 10]. Under this definition, NES differ from QES in which the allocation of exposure, whether partly controlled by the researcher or not, does not clearly resemble a random process.

A third distinction between QES and NES has been made that argues that NES describe the study of unplanned events whereas QES describe evaluations of events that are planned (but not controlled by the researcher), such as policies or programmes specifically aimed at influencing an outcome (Fig. 1c) [17]. In practice however, the distinction between these can be ambiguous.

When the assignment of exposure is not controlled by the researcher, with rare exceptions (for example lottery-system [22] or military draft [23] allocations), it is typically very difficult to prove that true (as-if) randomization occurred. Because of the ambiguity of ‘as-if randomization’ and the fact that the tools to assess this are the same as those used for assessment of internal validity in any observational study [12], the UK Medical Research Council (MRC) guidance advocates a broader conceptualisation of a NES. Under the MRC guidance, a NES is defined as any study that investigates an event that is not under the control of the research team, and which divides a population into exposed and unexposed groups, or into groups with different levels of exposure (Fig. 1d).

Here, while acknowledging the remaining ambiguity regarding the precise definition of a NES, in consideration of the definitions above [24], we argue that:

- what distinguishes NES from RCTs is that allocation is not controlled by the researchers and;
- what distinguishes NES from other observational designs is that they specifically evaluate the impact of a clearly defined event or process which result in differences in exposure between groups.

A detailed assessment of the allocation mechanism (which determines exposure status) is essential. If we can demonstrate that the allocation process approximates a randomization process, any causal claims from NES will be substantially strengthened. The plausibility of the ‘as-if random’ assumption strongly depends on detailed knowledge of why and how individuals or groups of individuals were assigned to conditions and how the assignment process was implemented [10]. This plausibility can be assessed quantitatively for observed factors using standard tools for assessment of internal validity of a study [12], and should ideally be supplemented by a qualitative description of the assignment process. Common with contemporary public health practice, we will use the term ‘natural experiment study’, or NES to refer to both NES and QES, from hereon.

## Methods

Medline, Embase and Google Scholar were searched using search terms including quasi-experiment, natural experiment, policy evaluation and public health evaluation and key methodological papers were used to develop this work. Peer-reviewed papers were supplemented by grey literature.

## Results

### Part 1. Conceptualisations of natural experiments

#### An analytic approach

Some conceptualisations of NES place their emphasis on the analytic tools that are used to evaluate natural experiments [25, 26]. In this conceptualisation NES are understood as being defined by the way in which they are analysed, rather than by their design. An array of different statistical methods is available to analyse natural experiments, including regression adjustments, propensity scores, difference-in-differences, interrupted time series, regression discontinuity, synthetic controls, and instrumental variables. Overviews including strengths and limitations of the different methods are provided in [12, 27]. However, an important drawback of this conceptualisation is that it suggests that there is a distinct set of methods for the analysis of NES.

#### A study design

The popularity of NES has resulted in some conceptual stretching, where the label is applied to a research design that only implausibly meets the definitional features of a NES [10]. For example, observational studies exploring variation in exposures (rather than the study of an event or change in exposure) have sometimes also been badged as NES. A more stringent classification of NES as a type of study design, rather than a collection of analytic tools, is important because it prevents attempts to incorrectly cover observational studies with a ‘glow of experimental legitimacy’ [10]. If the design rather than the statistical methodology defines a NES, this allows an open-ended array of statistical tools. These tools are not necessarily constrained by those mentioned above, but could also, for example, include new methods such as synthetic controls that can be utilised to analyse the natural experiments. The choice of appropriate evaluation method should be based on what is most suitable for each particular study, and then depends on the knowledge about the event, the availability of data, and design elements such as its allocation process.

Dunning argues that it is the overall research design, rather than just the statistical methods, that compels conviction when making causal claims. He proposes an evaluation framework for NES along the three dimensions of (1) the plausibility of as-if randomization of treatment, (2) the credibility of causal and statistical models, and (3) the substantive relevance of the treatment. Here, the first dimension is considered key for distinguishing NES from other QES [4]. NES can be divided into those where a plausible case for ‘as-if random’ assignment can be made (which he defines as NES), and those where confounding from observed factors is directly adjusted for through statistical means. The validity of the latter (which Dunning defines as ‘other quasi experiments’, and we define as ‘weaker NES’) relies on the assumption that unmeasured confounding is absent [8], and is considered less credible in theory for making causal claims [4]. In this framework, the ‘as-if-randomised’ NES can be viewed as offering stronger causal evidence than other quasi-experiments. In principle, they offer an opportunity for direct estimates of effects (akin to RCTs) where control for confounding factors would not necessarily be required [4], rather than relying on adjustment to derive conditional effect estimates [10]. Of course, the latter may well reach valid and compelling conclusions as well, but causal claims suffer to a higher degree from the familiar threats of bias and unmeasured confounding.

### Part 2. A target trial framework for natural experiment studies

In this section, we provide recommendations for evaluation of the ‘as if random’ assumption and provide a unifying Target Trial Framework for NES, which brings together key sets of criteria that can be used to appraise the strength of causal claims from NES and assist with study design and reporting.

In public health, there is considerable overlap between analytic and design-based uses of the term NES. Nevertheless, we argue that if we consider NES a type of study design, causal inference can be strengthened by clear appraisal of the likelihood of ‘as-if’ random allocation of exposure. This should be demonstrated by both empirical evidence and by knowledge and reasoning about the causal question and substantive domain under question [8, 10]. Because the concept of ‘as-if’ randomization is difficult, if not impossible to prove, it should be thought of along a ‘continuum of plausibility’ [10]. Specifically, for claims of ‘as-if’ randomization to be plausible, it must be demonstrated that the variables that determine treatment assignment are exogenous. This means that they are: i) strongly correlated with treatment status but are not caused by the outcome of interest (i.e. no reverse causality) and ii) independent of any other (measured or unmeasured) causes of the outcome of interest [8].

Given this additional layer of justification, especially with respect to the qualitative knowledge of the assignment process and domain knowledge from practitioners more broadly, we argue where feasible for the involvement of practitioners. This could, for example, be formalized through co-production in which members of the public and policy makers are involved in the development of the evaluation. If we appraise NES as a type of study design, which distinguish themselves from other designs because i) there is a particular change in exposure that is evaluated and ii) causal claims are supported by an argument of the plausibility of as-if randomization, then we guard against conflating NES with other observational designs [10, 28].

There is a range of ways of dealing with the problems of selection on measured and unmeasured confounders in NES [8, 10] which can be understood in terms of a ‘target trial’ we are trying to emulate, had randomization been possible [29]. The protocol of a target trial describes seven components common to RCTs (‘eligibility criteria’, ‘treatment strategies’, ‘assignment procedures’, ‘follow-up period’, ‘outcome’, ‘causal contrasts of interest’, and the ‘analysis plan’), and provides a systematic way of improving, reporting and appraising NES relative to a ‘gold standard’ (but often not feasible in practice) trial. In the design phase of a NES deviations from the target trial in each domain can be used to evaluate where improvements and where concessions will have to be made. This same approach can be used to appraise existing NES. The target trial framework also provides a structured way for reporting NES, which will facilitate evaluation of the strength of NES, improve consistency and completeness of reporting, and benefit evidence syntheses.

In Table 1, we bring together elements of the Target Trial framework and conceptualisations of NES to derive a framework to describe the Target Trial for NES [12]. By encouraging researchers to address the questions in Table 1, the framework provides a structured approach to the design, reporting and evaluation of NES across the seven target trial domains. Table 1 also provides recommendations to improve the strength of causal claims from NES, focussing primarily on sensitivity analyses to improve internal validity.

**Table 1:** Outline of the Target Trial Framework for Natural Experiment Studies

Protocol Component [29]	Theorising the causal contrast*	Strengthening causal claims*
Eligibility Criteria	• Does the study include a precise and detailed description of the population who have/will feasibly be exposed to the intervention, with special focus on the boundaries of the intervention which may be fuzzy and/or may not overlap with boundaries of (routine) data collection or risk of the outcome? • Is a definition and description of the eligibility of potential control populations to ensure independence and exclude spill-over effects included? [30] • Are potential issues of collider bias [31] or other forms of selection bias considered?	• Consider broadening out the eligibility criteria for multiple control groups that differ in some consequential way [14]; to include, for example, comparable groups or areas from other geographical locations for sensitivity analyses.
Treatment strategies	• Are the intervention, the dose and treatment regimes, and what it aims to affect, including when and where it is introduced defined? • Has the baseline timepoint been defined? • Has the control condition (including the potential for reactions even if intervention was not received) in the post-intervention period been defined, and/or has the counterfactual been defined? • Does the study describe the plausibility of the Stable Unit Treatment Value Assumption (SUTVA)? [32]	• Consider the possibility of pre-implementation changes resulting from anticipating the intervention (for example changes in behaviour or reactions from industry [33]). • Consider additional other, likely earlier, baseline timepoints to exclude anticipation behaviour in sensitivity analyses.
Assignment procedures	• Given that the assignment procedure of the intervention is not controlled by the researcher, has the assignment rationale and procedures been reported in detail? Note that the intervention group can also be the whole population (e.g. if exposed to the intervention at a well-defined timepoint). Further note that, in the absence of a suitable control population defined by a temporal or spatial boundary, that the control group can be a synthetic counterfactual • Has the plausibility of *as-if* randomization of the assignment been discussed? • Has conditional exchangeability been formally evaluated for observed factors? Note that this cannot be done for unobserved factors and requires knowledge about exposure allocation procedures. • Has the parallel trends assumption been assessed prior to the intervention implementation (when analysis based on timeseries data)? • Has the plausibility of intervention and control groups remaining in their allocation group throughout the study been discussed?	• Consider whether partial control of assignment of intervention is possible. • Consider the selection of controls that are geographically locally to the intervention units • Consider selection of intact control groups that are matched to intervention units based on pre-intervention measures of the outcome • Consider control groups for whom measurement of the exposure, outcome, and covariates is performed similarly to that for the intervention group [6]. • Consider inclusion of (additional) control groups or use of synthetic counterfactuals to improve assessment of conditional exchangeability for observed and unobserved factors [14]. • Consider the inclusion of additional controls hypothesized to not be affected by the intervention (negative controls)
Follow-up period	• Has the follow-up period, which starts prior to assignment of intervention to groups, includes assignment, and ends after a priori defined period post-intervention, been described?	• Consider different follow-up periods to assess evidence of pulse impacts (short-term temporal effect followed by regression to the mean)
Outcome(s)	• Does the study describe the outcome (or outcomes) of interest in detail, and does the description include a priori hypothesized individual-level or population-level parameters at *a priori* defined period post-intervention or cumulative/average outcomes from start of intervention until *a priori* defined period post-intervention?	Consider evaluation of additional outcomes: • also hypothesised to be affected by intervention (positive control) • hypothesised to be unaffected by intervention (negative control)
Causal contrasts of interest	• Has the causal contrast, or contrasts, to be evaluated been precisely defined? • Has the causal contrast of interest been specified as an ‘average-treatment-effect’ (ATE) for the population, or as ‘average-treatment-effect-treated’ (ATT) for self-selected interventions? [34]	• Consider, and report, whether Natural Experiment Study enables the estimation of intention-to-treat effects and/or per-protocol effects (although in natural experiments the latter may be rarely available) • Consider additional causal contrasts, for example in subgroups
Analysis plan	• Is there a pre-specified analytic plan? • Is the measure of the result specified as a relative and/or absolute measure? • Is the measure of the result specified as the difference between post-intervention minus pre-intervention outcome of interest in intervention group and post-intervention minus pre-intervention outcome of interest in control group? • Has the statistical methodology used to calculate the impact or effect of the event or intervention been described in sufficient detail to allow replication?	• Consider the inclusion of temporal falsification analyses by choosing different, randomly assigned, implementation times for the intervention • Consider the inclusion of spatial falsification analyses using different combinations of units, irrespective of true assignments • Consider improving causal claims by methodological triangulation using different statistical methods [35, 36].

*: Sources [1, 4, 37, 38] (unless otherwise indicated)

An illustrative example of a well-developed NES based on the criteria outlined in Table 1 is by Reeves et al. [39]. The NES evaluates the impact of the introduction of a National Minimum Wage on mental health. The study compared a clearly defined intervention group of recipients of a wage increase up to 110% of pre-intervention wage with clearly defined control groups of (1) people ineligible to the intervention because their wage at baseline was just above (100–110%) minimum wage and (2) people who were eligible, but whose companies did not comply and did not increase minimum wage. This study also included several sensitivity tests to strengthen causal arguments. We have aligned this study to the Target Trial framework in Additional file 1.

## Discussion

The Target Trial Approach for NES (outlined in Table 1) provides a straightforward approach to improve, report, and appraise existing NES and to assist in the design of future studies. It focusses on structural design elements and goes beyond the use of quantitative tools alone to assess internal validity [12]. This work complements the ROBINS-I tool for assessing risk of bias in non-randomised studies of interventions, which similarly adopted the Target Trial framework [40]. Our approach focusses on the internal validity of a NES, with issues of construct and external validity being outside of the scope of this work (guidelines for these are provided in for example [41]). It should be acknowledged that less methodologically robust studies can still reach valid and compelling conclusions, even without resembling the notional target trial. However, we believe that drawing on the target trial framework helps highlight occasions when causal inference can be made more confidently.

And finally, the framework does explicitly exclude observational studies that aim to investigate the effects of changes in behaviour without an externally forced driver to do so. For example, although a cohort study can be the basis for the evaluation of a NES in principle, effects of the change of diet of some participants (compared to those who did not change their diet) is not an external cause (i.e. exogenous) and does not fall within the definition of an experiment [11]. However, such studies are likely to be more convincing than those which do not study within-person changes and we note that the statistical methods used may be similar to NES.

Despite their advantages, NES remain based on observational data and thus biases in assignment of the intervention can never be completely excluded (although for plausibly ‘as if randomised’ natural experiments these should be minimal). It is therefore important that a robust assessment of different potential sources of bias is reported. It has additionally been argued that sensitivity analyses are required to assess whether a pattern of small biases could explain away any ostensible effect of the intervention, because confidence intervals and statistical tests do not do this [14]. Recommendations that would improve the confidence with which we can make causal claims from NES, derived from work by Rosenbaum [14], have been outlined in Table 1. Although sensitivity analyses can place plausible limits on the size of the effects of hidden biases, because such analyses are susceptible to assumptions about the maximum size of omitted biases, they cannot completely rule out residual bias [34]. Of importance for the strength of causal claims therefore, is the triangulation of NES with other evaluations using different data or study designs susceptible to different sources of bias [5, 42].

None of the recommendations outlined in Table 1 will by themselves eliminate bias in a NES, but neither is it required to implement all of them to be able to make a causal claim with some confidence. Instead, a continuum of confidence in the causal claims based on the study design and the data is a more appropriate and practical approach [43]. Each sensitivity analysis aims to minimise ambiguity of a particular potential bias or biases, and as such a combination of selected sensitivity analyses can strengthen causal claims [14]. We would generally, but not strictly, consider a well conducted RCT as the design where we are most confident about such claims, followed by natural experiments, and then other observational studies; this would be an extension of the Grading of Recommendations Assessment, Development, and Evaluation (GRADE) framework [44]. GRADE provides a system for rating the quality (or certainty) of a body of evidence and grading the strength of recommendations for use in systematic reviews, health technology assessments (HTAs), and clinical practice guidelines. It typically only distinguishes between trials and observational studies when making these judgments (note however, that recent guidance does not make this explicit distinction when using ROBINS-I [45]). Given the increased contribution of NES in public health, especially those based on routine data [37], the specific inclusion of NES in this system might improve the rating of the evidence from these study designs.

Our recommendations are of particular importance for ensuring rigour in the context of (public) health research where natural experiments have become increasingly popular for a variety of reasons, including the availability of large routinely collected datasets [37]. Such datasets invite the discovery of natural experiments, even where the data may not be particularly applicable to this design, but also these enable many of the sensitivity analyses to be conducted from within the same dataset or through linkage to other routine datasets.

Finally, alignment to the Target Trial Framework also links natural experiment studies directly to other measures of trial validity, including pre-registration, reporting checklists, and evaluation through risk-of-bias-tools [40]. This aligns with previous recommendations to use established reporting guidelines such as STROBE, TREND [12], and TIDieR-PHP [46] for the reporting of natural experiment studies. These reporting guidelines could be customized to specific research areas (for example, as developed for a systematic review of quasi-experimental studies of prenatal alcohol use and birthweight and neurodevelopment [47]).

## Conclusions

We provide a conceptualisation of natural experiment studies as they apply to public health. We argue for the appreciation of natural experiments as a type of study design rather than a set of tools for the analyses of non-randomised interventions. Although there will always remain some ambiguity about the strength of causal claims, there are clear benefits to harnessing NES rather than relying purely on observational studies. This includes the fact that NES can be based on routinely available data and that timely evidence of real-world relevance can be generated. The inclusion of a discussion of the plausibility of as-if randomization of exposure allocation will provide further confidence in the strength of causal claims.

Aligning NES to the Target Trial framework will guard against conceptual stretching of these evaluations and ensure that the causal claims about whether public health interventions ‘work’ are based on evidence that is considered ‘good enough’ to inform public health action within a ‘practice-based evidence’ framework. This framework describes how evaluations can help reducing critical uncertainties and adjust the compass bearing of existing policy (in contrast to the ‘evidence-based practice’ framework in which RCTs are used to generate ‘definitive’ evidence for particular interventions) [48].

## Supplementary Information

**Additional file 1.** Online Supplementary Material. Table 1. the Target Trial for Natural Experiments and Reeves et al. [28]. Alignment of Reeves et al. (Introduction of a National Minimum Wage Reduced Depressive Symptoms in Low-Wage Workers: A Quasi-Natural Experiment in the UK. Heal Econ. 2017;26:639–55) to the Target Trial framework.

## Data Availability

Data sharing is not applicable to this article as no datasets were generated or analysed during the current study.

## Abbreviations

RCT: Randomised Controlled Trial
NE: Natural Experiment
SUTVA: Stable Unit Treatment Value Assumption
ITT: Intention-To-Treat
