# Supplementary material for: Conceptualising natural and quasi experiments in public health
Source: BMC Med Res Methodol. 2021 Feb 11;21:32. doi: 10.1186/s12874-021-01224-x (PMC7879679; doi:10.1186/s12874-021-01224-x)
Supplement: Supplementary file 1 — Additional file 1. Online Supplementary Material. Table 1. the Target Trial for Natural Experiments and Reeves et al. [28]. Alignment of Reeves et al. (Introduction of a National Minimum Wage Reduced Depressive Symptoms in Low-Wage Workers: A Quasi-Natural Experiment in the UK. Heal Econ. 2017;26:639–55) to the Target Trial framework. [file 12874_2021_1224_MOESM1_ESM.pdf]

CONCEPTUALISING NATURAL AND QUASI EXPERIMENTS IN PUBLIC HEALTH

Frank de Vocht[1,2,3], Srinivasa Vittal Katikireddi[4], Cheryl McQuire[1,2], Kate Tilling[1,5], Matthew Hickman[1], Peter Craig[4]

[1] Population Health Sciences, Bristol Medical School, University of Bristol. [2] NIHR School for Public Health Research. [3] NIHR Applied Research Collaboration West. [5] MRC IEU. [4] MRC/CSO Social and Public Health Sciences Unit, University of Glasgow

ONLINE SUPPLEMENTARY MATERIALS

This study is funded by the National Institute for Health Research (NIHR) School for Public Health Research (Grant Reference Number PD-SPH-2015). The views expressed are those of the author(s) and not necessarily those of the NIHR or the Department of Health and Social Care. The funder had no input in the writing of the manuscript or decision to submit for publication. The NIHR School for Public Health Research is a partnership between the Universities of Sheffield; Bristol; Cambridge; Imperial; and University College London; The London School for Hygiene and Tropical Medicine (LSHTM); LiLaC – a collaboration between the Universities of Liverpool and Lancaster; and Fuse - The Centre for Translational Research in Public Health a collaboration between Newcastle, Durham, Northumbria, Sunderland and Teesside Universities. FdV is partly funded by National Institute for Health Research Applied Research Collaboration West (NIHR ARC West) at University Hospitals Bristol NHS Foundation Trust. SVK and PC acknowledge funding from the Medical Research Council (MC_UU_12017/13) and Scottish Government Chief Scientist Office (SPHSU13 & SPHSU15). SVK acknowledges funding from a NRS Senior Clinical Fellowship (SCAF/15/02). KT works in the MRC Integrative Epidemiology Unit, which is supported by the Medical Research Council (MRC) and the University of Bristol [MC_UU_00011/3].

Table 1. the Target Trial for Natural Experiments and Reeves et al. [39]

**Protocol Component**	**Theorising the causal contrast**		**Strengthening causal claims**	
	**Consideration**	**Reported in Reeves et al.**	**Recommendation**	**Reported in Reeves et al.**
**Eligibility Criteria**	Does the study include a precise and detailed description of the population who have/will feasibly be exposed to the intervention, with special focus on the boundaries of the intervention which may be fuzzy and/or may not overlap with boundaries of (routine) data collection or risk of the outcome? .	The study population are subjects enrolled in the British Household Panel Survey. A nationally representative longitudinal survey of 5,500 households and ~10,000 individuals (More detail [39]), and includes men and women aged 22-59 who worked at least 1hr per week in 1998 and 1999. The intervention group were individuals who received the minimum wage increase and the control population were those who did not, either because they were ineligible or because their employer did not comply with the legislation.	Consider broadening out the eligibility criteria for multiple control groups that differ in some consequential way; to include, for example, comparable groups or areas from other geographical locations for sensitivity analyses.	A second control group was used in the study, and were people who were eligible, but whose companies did not comply with the National Minimum Wage and did not increase minimum wage. Both control groups were combined in some analyses
	Is a definition and description of the eligibility of potential control populations to ensure independence and exclude spill-over effects included?	Introducing a minimum wage may also increase wages for those who are just above the minimum wage threshold, and so, those who are untreated could also be influenced by the intervention. While such spill-over effects are theoretically plausible, and this appears to have occurred in the USA, previous studies suggest that		

		they did not influence the UK's wage distribution.		
	Are potential issues of collider bias or other forms of selection bias considered?	The intervention group was selected based on post-intervention wages, which may have created some selection bias because psychologically fragile people may be more likely to be exploited by their firms and not receive the National Minimum Wage. This was assessed in sensitivity analysis		
**Treatment strategies**	Are the intervention, the dose and treatment regimes, and what it aims to affect, including when and where it is introduced defined?	The intervention was the introduction of the UK National Minimum Wage brought into force on April 1st, 1999. This was aimed to affect all workers who earned less than £3.60 per hour in 1998.<br><br>The intervention group comprises those who earned less than £3.60 per hour in 1998 and who then earned between £3.60 and £4.00 per hour in 1999.	Consider the possibility of pre-implementation changes resulting from anticipating the intervention (for example changes in behaviour or reactions from industry).	
	Has the baseline timepoint been defined?	The baseline timepoint was wave 8 of the data collection, which started in September 1998.		
	Has the control condition (including the potential for reactions even if intervention wasn't received) in the post-intervention period been defined, and/or has the counterfactual been defined?	The control condition is people ineligible to the intervention because their wage at baseline was just above (100-110%, or (£3.60 to £4 per hour) the threshold to receive Minimum Wage.	Consider other, likely earlier, baseline timepoint to exclude anticipation behaviour in sensitivity analyses	Robustness analyses were conducted using standard regression methods using an earlier start point of 1994.

		A second control group was used in the study, and were people who were eligible, but whose companies did not comply with the National Minimum Wage legislation and did not increase minimum wage.		For reference: the policy was a key policy for Labour in the 1997 election and a key piece of legislation in 1998
	Does the study describe the plausibility of the Stable Unit Treatment Value Assumption (SUTVA)?			
**Assignment procedures**	Given that the assignment procedure of the intervention is not controlled by the researcher, has the assignment rationale and procedures been reported in detail?	The primary control group were people ineligible to the intervention because their wage at baseline was just above (100-110%) the threshold to receive Minimum Wage.	Consider whether partial control of assignment of intervention is possible	This was not possible. Retrospective analyses of routine data
	Has the plausibility of *as-if* randomisation of the assignment been discussed?	Selection in either group based on the arbitrary threshold is plausibly independent from other factors and can be considered exogenous, or as-if random (an instrumental variable (IV)).<br><br>The plausibility is less strong for control group 2 because allocation relies on compliance of companies.<br><br>This was examined for observed factors using statistical tests.	Consider the selection of controls that are geographically locally to the intervention units	
			Consider selection of intact control groups that are matched to intervention units based on pre-intervention measures of the outcome	No matching was performed
			Consider control groups for whom measurement of the exposure, outcome, and covariates is performed similarly to that for the intervention group	All data are obtained from the British Household Panel Survey
			Consider inclusion of (additional) control groups or use of synthetic counterfactuals to improve assessment of conditional	Two control groups were used. Conditional exchangeability was assessed for observed factors

			exchangeability for observed and unobserved factors	
	Has the parallel trends assumption been assessed prior to the intervention implementation (when analysis based on time series data)		Consider the inclusion of additional controls hypothesized to not be affected by the intervention (negative controls)	No comparable group unaffected by the intervention was identified
	Has the plausibility of intervention and control groups remaining in their allocation group throughout the study been discussed?	While in theory it is possible that persons above the income threshold could choose to move into a lower income group, this was judged very unlikely.<br>There was zero attrition from 1998 to 1999 in the analytic sample.		
	The intervention group can also be the whole population (e.g. if exposed to the intervention at a well-defined timepoint) or, in the absence of a suitable control population defined by a temporal or spatial boundary, can be a synthetic counterfactual.			
**Follow-up period**	Has the follow-up period, which starts prior to assignment of intervention to groups, includes assignment, and ends after *a priori* defined period post-intervention, been described?	Follow-up starts at wave 8 of the data collection (1998), and post-intervention data are for wave 9 (start September 1999).	Consider different follow-up periods to assess evidence of pulse impacts (short-term temporal effect followed by regression to the mean)	Assessed whether positive mental health effects were sustained by adding a subsequent year, 2000/2001
**Outcome**	Does the study describe the outcome (or outcomes) of interest in detail, and does the description include *a priori* hypothesized individual-level or population-level parameters at *a priori* defined period post-intervention or cumulative/average outcomes from start of intervention until *a priori* defined period post-intervention?	The primary outcome was the probability of having a mental health problem assessed using the General Health Questionnaire 12 item version (GHQ-12), analysed as a continuous variable.	Consider evaluation of additional outcomes also hypothesised to be affected by intervention (positive control)	Additional outcomes were (from GHQ-12):<br>• ‘constantly under strain’<br>• ‘unhappy or depressed’<br>• self-reported depression<br><br>Additional outcomes also sensitive to short-term fluctuations in outcomes:

				• self-reported elevated blood pressure<br>• number of cigarettes smoker per day among current smokers<br><br>Analyses were repeated with different calculations of the outcome:<br>• different overtime premiums<br>• a wage gap estimator
			Consider evaluation of additional outcomes unaffected by intervention (negative control)	Additional outcomes hypothesised to be unaffected by intervention were (from GHQ-12)<br>Self-reported chronic conditions (e.g. hearing difficulties)
**Causal contrasts of interest**	Has the causal contrast, or contrasts, to be evaluated been precisely defined?	Change in GHQ-12 total scores from 1998 to 1999, specific components of the GHQ-12 (feelings of being ‘constantly under strain’ or ‘unhappy or depressed’ and a BHPS measure of self-reported depression between the intervention and control group.	Consider, and report, whether Natural Experiment Study enables the estimation of intention-to-treat effects and/or per-protocol effects (although in natural experiments the latter may be rarely available)	The study was considered a per-protocol design. If someone earned below the threshold they would receive the new Minimum Wage (in the intervention group), unless their employer did not comply, while higher increases would indicate wage increases through other means
			Consider other causal contrasts, for example in subgroups	Additional analyses were conducted for low-wage workers earning below the median (<£6 per hour) and those above the median (£6-£10 per hour).<br><br>Analyses were conducted after removing of people with second jobs.<br><br>Analyses were conducted after removing people who may have been earning below the minimum wage because of an exemption
	Has the causal contrast of interest been specified as an ‘average-treatment-effect’ (ATE) for the population, or as ‘average-treatment-effect-treated’ (ATT) for self-selected interventions?	The average intervention effect (ATE) was evaluated		

**Analysis plan**	Is there a pre-specified analytic plan?		Consider the inclusion of temporal falsification analyses by choosing different, randomly assigned, implementation times for the intervention	Estimated a series of random-effects<br><br>linear regression models (controlling for time dummies) that include observations from 1994 to 2001
	Is the measure of the result specified as a relative and/or absolute measure?			
	Is the measure of the result specified as the difference between post-intervention minus pre-intervention outcome of interest in intervention group and post-intervention minus pre-intervention outcome of interest in control group?	Difference-in-difference modelling framework.<br><br>Both differenced models and fixed-effects regression models including an interaction term between a period dummy and an intervention indicator.<br><br>Models were adjusted for age, sex, social class, and education.	Consider the inclusion of spatial falsification analyses using different combinations of units, irrespective of true assignments	
	Has the statistical methodology used to calculate the impact or effect of the event or intervention been described in sufficient detail to allow replication?		Consider improving causal claims by methodological triangulation using different statistical methods	Analyses were repeated with the natural log of the dependent variable<br><br>Natural experiment results were compared with traditional multivariable regression models.
